# DBC1/CCAR2 and CCAR1 Are Largely Disordered Proteins that Have Evolved from One Common Ancestor

**DOI:** 10.1155/2014/418458

**Published:** 2014-12-11

**Authors:** Jessica Brunquell, Jia Yuan, Aqeela Erwin, Sandy D. Westerheide, Bin Xue

**Affiliations:** Department of Cell Biology, Microbiology, and Molecular Biology, College of Arts and Sciences, University of South Florida, 4202 East Fowler Avenue, ISA 2015, Tampa, FL 33620, USA

## Abstract

Deleted in breast cancer 1 (DBC1, CCAR2, KIAA1967) is a large, predominantly nuclear, multidomain protein that modulates gene expression by inhibiting several epigenetic modifiers, including the deacetylases SIRT1 and HDAC3, and the methyltransferase SUV39H1. DBC1 shares many highly conserved protein domains with its paralog cell cycle and apoptosis regulator 1 (CCAR1, CARP-1). In this study, we examined the full-length sequential and structural properties of DBC1 and CCAR1 from multiple species and correlated these properties with evolution. Our data shows that the conserved domains shared between DBC1 and CCAR1 have similar domain structures, as well as similar patterns of predicted disorder in less-conserved intrinsically disordered regions. Our analysis indicates similarities between DBC1, CCAR1, and the nematode protein lateral signaling target 3 (LST-3), suggesting that DBC1 and CCAR1 may have evolved from LST-3. Our data also suggests that DBC1 emerged later in evolution than CCAR1. DBC1 contains regions that show less conservation across species as compared to the same regions in CCAR1, suggesting a continuously evolving scenario for DBC1. Overall, this study provides insight into the structure and evolution of DBC1 and CCAR1, which may impact future studies on the biological functions of these proteins.

## 1. Introduction

DBC1 (deleted in breast cancer 1, KIAA1967, CCAR2) and paralog CCAR1 (cell cycle and apoptosis regulator 1, CARP1) are emerging as important regulators for a variety of physiological processes. DBC1 was originally identified by its localization to a region of chromosome 8p21 that is homozygously deleted in breast cancer. However, DBC1 is not localized to the epicenter of the deletion and is therefore not the strongest candidate tumor suppressor gene in this region [1, 2]. DBC1 is now also called CCAR2 (cell cycle and apoptosis regulator 2) in order to distinguish it from an unrelated protein that is also named DBC1 (deleted in bladder cancer 1) [3]. DBC1 exerts some of its biological effects through interactions with protein modifying enzymes, including the deacetylases SIRT1 and HDAC3, and the methyltransferase SUV39H1 [4–7]. Through its many interactions, DBC1 regulates a variety of cellular processes including aging, metabolism, apoptosis, and stress response pathways [4, 7–11]. DBC1 studies are currently expanding to uncover new interacting partners and the possibility of roles in other biological processes.

CCAR1 is the paralog to DBC1 that was originally identified as a mediator of apoptosis in a process that involves sequestration of 14-3-3 and altered expression of multiple cell cycle regulatory genes [8, 9, 12]. CCAR1 can bind to the mediator complex and enhance transcription of estrogen receptor and glucocorticoid receptor target genes and can act as a coactivator for p53-dependent transcription [13]. CCAR1 can also cooperatively bind to DBC1 and synergistically enhance estrogen receptor function [14]. Thus, like DBC1, CCAR1 is also involved in a variety of cellular processes and functions together with DBC1 in some cases.

DBC1 and CCAR1 share many of the same functional domains including an S1-Like domain and a nuclear localization signal (NLS) on the N-terminus, a Leucine zipper (LZ) domain and a Nudix domain that are centrally located, and an EF-Hand domain and coiled-coil segments on the C-terminus [9, 14, 15]. Experimental evidence regarding the specific functions of the S1-Like, Nudix, and EF-Hand domains for both DBC1 and CCAR1 have not yet been determined. CCAR1 (1150aa) is a larger protein compared to DBC1 (923aa) due to the presence of two extra domains, including a centrally located SAP domain and an extra coiled-coil segment found immediately after the SAP domain.

The N-terminus of DBC1 (aa1–264) is the region where most of the currently known protein-protein interactions have been mapped. The S1-Like domain was originally identified in the ribosomal protein S1. Proteins that contain homology to this domain typically have RNA binding capabilities, suggesting evolution from an ancient nucleic acid binding protein [16]. The NLS is an important site for regulation via post-translational modifications, where acetylation can disrupt DBC1 translocation into the nucleus and ultimately inhibit nuclear interactions [17]. The LZ is a structural motif that functions as a dimerization domain and can bind to DNA to regulate gene expression in DBC1, but it is likely non-functional in CCAR1 [18]. DBC1 interactions with epigenetic modifiers, nuclear receptors, and mRNA splicing components all take place within the N-terminal area [4–7, 14, 17]. The DBC1/SIRT1 interaction has been highly studied due to the important role that DBC1 plays in inhibiting the epigenetic modifications that are regulated by SIRT1. Conflicting data points to either the LZ of DBC1 (aa243–264) [4, 14, 19, 20] or the N-terminal amino acids 1–240 as being critical for this interaction with SIRT1 [17].

The central region of DBC1 and CCAR1 contains a Nudix domain that is catalytically inactive due to the absence of key amino acid residues within the catalytic site. However, it has been suggested to play a role in sensing the products of the SIRT1 deacetylase reaction [9]. The SAP domain, specific to CCAR1, is also centrally located. This domain shares homology with a DNA binding motif commonly found in a diverse set of nuclear proteins that are typically involved in chromosomal organization [21].

The C-terminus of both DBC1 and CCAR1 contains an EF-Hand domain. EF-Hand domains bind to calcium ions and regulate gene expression but, similar to the Nudix domain, the EF-Hand of DBC1 and CCAR1 may not be functional because it is unlikely to bind to calcium ions [8]. Both proteins also contain a coiled-coil segment in the C-terminus, with an extra coiled-coil segment present in CCAR1. Coiled-coil regions are known to contain important protein interaction motifs [22]. The coiled-coil region of DBC1 has been shown to interact with only one protein thus far, the circadian cycle nuclear receptor Rev-erb*α*. Overexpression of DBC1 can enhance the stability and expression of Rev-erb*α*, ultimately affecting circadian oscillations and metabolism [23].

Evaluating the detailed evolutionary path that DBC1 and CCAR1 have taken, and the factors that have influenced the evolutionary path, will expand our knowledge base of these two proteins. The presence of highly conserved domains shared between DBC1 and CCAR1 indicates that DBC1 and CCAR1 may have evolved from one common ancestor. Corresponding to this, an evolutionary connection between DBC1 and CCAR1 has been reported in the large ortholog database OrthoDB [24]. Interestingly, DBC1 and CCAR1 are predicted to be intrinsically disordered as demonstrated in the D2P2 database of predicted disordered proteins [25]. As shown by previous studies on other proteins [26–36], evolutionary analysis that takes protein intrinsic disorder into account is particularly informative. Intrinsically disordered regions within proteins can be critical for protein function, as these regions are structurally flexible and are frequent sites of protein interactions and various modifications [37–40]. In many cases, intrinsically disordered regions are also associated with higher substitution rates [32, 41, 42].

In this study, we have predicted the structural properties of DBC1 and CCAR1, and our findings support the function of both proteins in many protein-protein interactions due to the high occurrence of disordered residues. Phylogenetic analysis predicts that both DBC1 and CCAR1 evolved from one common ancestor, the nematode protein LST-3. Collectively, this study provides insight into the future studies of DBC1 and CCAR1 by evaluating both evolution and structure.

## 2. Materials and Methods

### 2.1. DBC1 Homologs and Paralogs

BLASTP [43, 44] was used to align the human DBC1 (hDBC1) protein sequence (UniProt ID: Q8N163) against the entire UniProt database [45]. The obtained UniProt hits were filtered using a cutoff alignment score set at 10% of the hDBC1-hDBC1 alignment score based on trial and error. The 129 sequences that remained had a minimal sequence similarity of ~30% and a minimal sequence coverage of ~50% as compared to hDBC1, complying with previous studies [42]. After removing redundant sequences using BLASTClust and cutoff of 90% sequence identity, 93 sequences from 65 species were used for analysis (Table  S1; Supplementary Material available online at http://dx.doi.org/10.1155/2014/418458). The 65 species include mammals, birds, reptiles, amphibians, fish, insects, and nematodes. The proteins found include DBC1, CCAR1, LST-3, and other undefined generic names. In addition, the complete proteomes of human, zebrafish, and* C. elegans* were downloaded from UniProt for comparative studies.

### 2.2. Disorder Prediction

PONDR-FIT [46] and PONDR-VLXT [47] were employed to run disorder prediction analysis. The disorder scores from both PONDR-FIT and PONDR-VLXT were used to measure the flexibility of the amino acids in all of the proteins examined. PONDR-FIT is one of the most accurate disorder predictors as it adopts the metapredictor strategy. Metapredictors are prevailing in the field of disorder prediction due to improved accuracy [46, 48]. Many state-of-the-art disorder predictors are metapredictors [48–53]. PONDR-FIT uses an artificial neural network to optimize the prediction results from six component predictors: PONDR-VLXT [47], PONDR-VSL2 [54, 55], PONDR-VL3 [56, 57], FoldIndex [58], IUPred [59], and TopIDP [60]. PONDR-FIT improves the prediction accuracy significantly in various testing datasets compared to its component predictors [46]. PONDR-VLXT is the first generation of disorder prediction software that is specifically designed to detect local flexibility of amino acid sequences. Although PONDR-VLXT is not the most accurate tool, it is still powerful due to its sensitivity to amino acid composition [46, 61]. PONDR-VLXT has been successfully applied in detecting linear interaction motifs (MoRFs) [62–64], which have proven to be extremely abundant in protein-protein interactions [65–68]. The values of predicted disorder scores were applied to measure the structural flexibility of proteins and the combination of disorder scores from PONDR-VLXT, PONDR-FIT, and other predictors, has been applied in many studies in order to explore a broad range of biological questions. Examples of these studies include methionine oxidation [69], phosphorylation [70], p53 evolution [42], binding motifs [71], iPS transcription factors [72], PTEN interactions [70], the spliceosome [73], the structural flexibility of viral proteins [72], and evolution across species [74, 75]. In this paper, we have also used predicted disorder scores to measure the structural flexibility of whole protein sequences along with local regions.

### 2.3. CH-CDF Analysis

The previously mentioned predictors were used to predict intrinsic disorder at a residue level. Intrinsic disorder can also be measured at the entire sequence level using a Charge-Hydropathy and Cumulative Distribution Function (CH-CDF) plot [51, 76]. A CH-CDF plot is composed using parameters from both a Charge-Hydropathy (CH) plot [77] and a Cumulative Distribution Function (CDF) plot [78]. In each of these two plots, structured proteins and disordered proteins stay in different regions and can be separated by a linear boundary line. The distance of a protein from the boundary line in each of them (dCH in CH plot and dCDF in CDF plot) describes the tendency of the protein being structured or disordered. The sign of the distance (positive or negative) shows whether the entire protein is structured or disordered. The performances of these two individual plots are often complementary. Therefore, their combination improves the prediction accuracy at the sequential level to 90% [51, 76]. In the CH-CDF plot, the directional distance dCH in the CH plot is set as the *y*-axis, and the directional distance dCDF in the CDF plot is used as the *x*-axis. As dCH and dCDF both have positive and negative values, the entire CH-CDF plot can be split into four quadrants using dCH = 0 and dCDF = 0: (1) Q1, dCH ≥ 0 & dCDF ≥ 0; (2) Q2, dCH < 0 & dCDF ≥ 0; (3) Q3, dCH < 0 & dCDF < 0; and (4) Q4, dCH ≥ 0 & dCDF < 0. By definition, proteins in Q2 are predicted to be structured. Proteins in Q3 and Q4 are disordered. Proteins in Q1 have excessive charged residues but can be structured.

### 2.4. Three-Dimensional Structure Prediction

The 3D structure of the structured domains for both human DBC1 and CCAR1 were built using HHpred [79], RaptorX [80], and I-TASSER [81]. Each structured domain has three predicted structures. The structure with the highest QMean score [82] was selected as the final predicted structure.

### 2.5. Phylogenetic Analysis

Mega5 [83] was used to run multiple sequence alignments and to analyze the phylogeny of the sequences. CLUSTALW was chosen to perform the multiple sequence alignment (default PAM matrix, Gap opening score 10, and Gap extension score 0.1). Nearest-neighboring algorithm was selected to analyze the phylogenetic relations among the sequences. The final phylogenetic tree was obtained by bootstrapping 2000 times.

### 2.6. Genome Neighborhood Analysis

We calculated the Conservation of Genomic Neighborhood (CGN) score [84, 85] of both DBC1 and CCAR1 for selected species, including mammals, birds, insects, reptiles, amphibians, and fish. When calculating the CGN score, all of the genes within a window of two million bases from the center of DBC1 or CCAR1 of that species were extracted from GeneBank. The number of total genes in the window of the human genome was defined as *M*_HS_, the number of common genes between human and another species *X* was counted as *C*_*X*_, and then the CGN score of species *X* was calculated as CGN_*X*_ = *C*_*X*_/*M*_HS_. The human and mouse gene lists were also used to build a synteny plot [86–88].

### 2.7. Mutation Rate Analysis

The substitution rate between each group of domains between each species set is calculated as follows. (1) Two groups of sequences were aligned using CLUSTALW. (2) The domain structure of hCCAR1 was used to label the location of similar regions in all of the other sequences. (3) The amino acid of each sequence, on each site within a specific region, in the second group was compared with the amino acid sequence in the first group. If no match was found, a substitution was recorded for this site. (4) This process was repeated for each group with step (3) for all of the sequences in the second group. The final substitution frequency is *S*_*i*_ for the *i*th site. (5) The sum was calculated for all of the substitutions for the sites in the entire region to get the total substitution ∑*S*_*i*_  (*i* = 1,…, *L*), where *L* is the number of sites in this region. (6) For example, if there were *M* sequences in the first group and *N* sequences in the second group, the final substitution rate would be ∑*S*_*i*_/(*M*∗*N*∗*L*).

## 3. Results

### 3.1. DBC1 and CCAR1 Are Intrinsically Disordered Proteins with a Similar Domain Structure and a Similar Pattern of Predicted Intrinsic Disorder

Human DBC1 (hDBC1) and human CCAR1 (hCCAR1) have ~30% sequence similarity to each other (Figure  S1) and share multiple highly conserved domains.  Table 1 shows the sequential locations of the functional domains for both proteins and indicates the known or predicted functions of the domains. The sequence alignment of each domain is provided in Figures  S2 and  S3. The size difference between DBC1 and CCAR1 is the result of three segments found in hCCAR1 that hDBC1 lacks, including an elongated N-terminal disordered region, a SAP domain, and an extra coiled-coil segment in its C-terminus (CC1).

**Figure 1:**
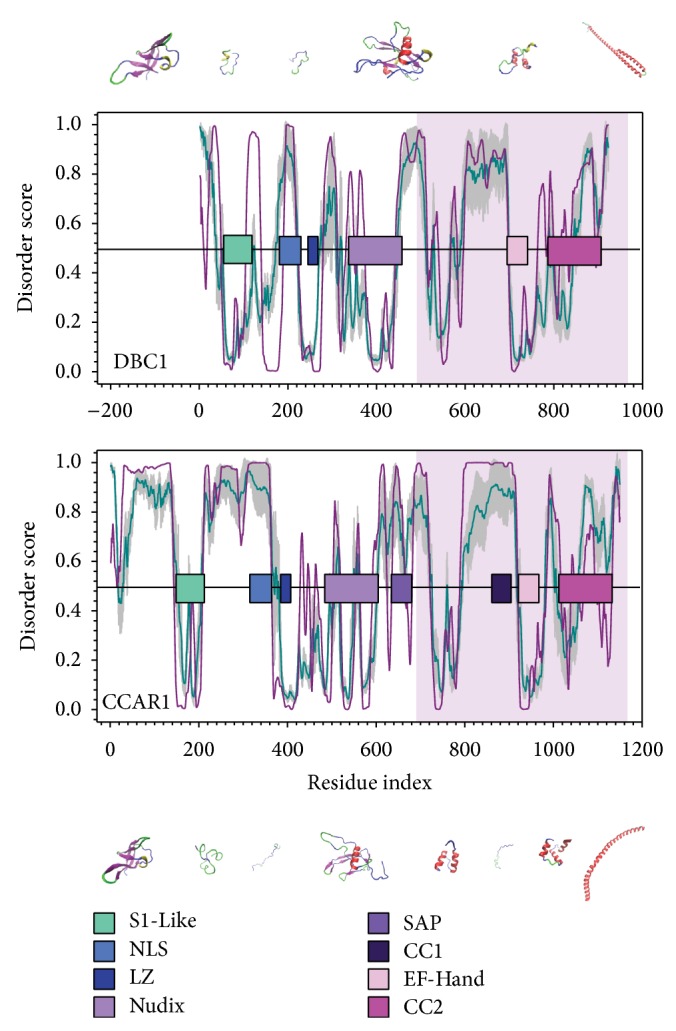
Disorder analysis shows the domain structure and molecular flexibility of hDBC1 and hCCAR1. The curves present the disorder score predicted by PONDR-FIT (FIT, dark cyan) and PONDR-VLXT (VLXT, dark pink). The *x*-axis of human DBC1 (hDBC1, UniProtID: Q8N163) is shifted by 200 residues in order to align the C-terminus to human CCAR1 (hCCAR1, UniProtID: Q8IX12). The gray shadow behind PONDR-FIT represents the prediction of error. Residues with a score higher than 0.5 are disordered, while residues with a score lower than 0.5 are structured. The horizontal bars are the conserved functional domains identified in both proteins (S1-Like: aqua blue; NLS: medium blue; LZ: dark blue; Nudix: light purple; SAP: medium purple; CC1: dark purple; EF-Hand: light pink; CC2: dark pink). The predicted 3D structures are scaled roughly with their lengths.

In order to compare the similarities in conformational properties between hDBC1 and hCCAR1, the degree of predicted protein intrinsic disorder of these two proteins was analyzed (Figure 1). While 41% of hDBC1 is composed of disordered residues, 61% of residues are disordered in hCCAR1. As expected, a majority of the functional domains listed in Table 1, including the S1-Like, LZ, Nudix, and EF-Hand, are located in the structured segments of both proteins. The domains that are intrinsically disordered, or have a high degree of structural flexibility as indicated by higher disorder score, are the NLS and coiled-coil segments on hDBC1 and hCCAR1, as well as the SAP domain that is specific to hCCAR1. The predicted 3D structures of these functional domains indicate that many of these domains are structured and have limited flexibility.

Analysis of the disorder curves on the C-terminal residues (~400aa) of hDBC1 and hCCAR1 indicates a high degree of similarity on the curve of predicted protein intrinsic disorder in this region, thereby suggesting a similar conformational fluctuation and further functional role (Figure 1, shaded region). Corresponding to this finding, a sequence alignment between these two regions shows 30% sequence identity, indicating evolutionary conservation (Figure  S3). The high degree of similarity between the structure of the conserved domains in hDBC1 and hCCAR1, along with the similarity in the C-terminal region, leads to the presumption that hDBC1 and hCCAR1 may share a common molecular origin.

**Figure 2:**
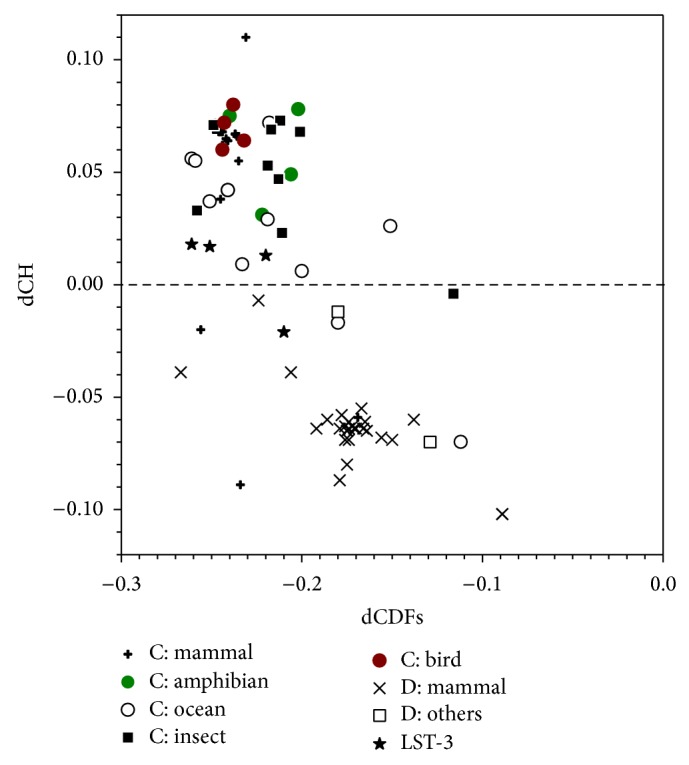
CH-CDF analysis for all DBC1 and CCAR1 proteins. The *x*-axis and *y*-axis are the CDF and CH distances, respectively. The CDF distance is calculated from PONDR-VSL2 prediction. All DBC1 proteins are split into two groups: mammal and others (starts with “D” in the legend). All CCAR1 proteins are arranged into six groups, including mammal, amphibian, aquatic animals, insect, and bird (as denoted by “C” in the legend). Nematode LST-3 proteins are in one group.

In addition to hDBC1 and hCCAR1, we determined the sequence level of intrinsic disorder from other species using a CH-CDF plot. We carried out CH-CDF analysis for all of the proteins in our dataset and found that all DBC1 and CCAR1 proteins have large negative values (<−0.1) on CDF distance, indicating that these proteins are mostly intrinsically disordered (Figure 2). In terms of CH distances, CCAR1 and DBC1 proteins have varying distributions. Most CCAR1 proteins have a positive CH distance, while all DBC1 proteins have a negative CH distance. Detailed analysis indicates that many groups have localized distribution on this CH-CDF plot, such as amphibian CCAR1 and bird CCAR1. Mammalian CCAR1 proteins have the broadest distribution on CH distance, followed by CCAR1 proteins from aquatic animals. Compared to mammalian DBC1 proteins, mammalian CCAR1 proteins are more scattered in the CH-CDF plot, indicating more structural variability. Therefore, based on the algorithms of CH and CDF distance, it can be concluded that most CCAR1 proteins have extra charged residues, while almost all DBC1 proteins are more structure-prone.

### 3.2. Human DBC1 Shares Common Ancestry with the Nematode CCAR1 Ortholog LST-3

**Figure 3:**
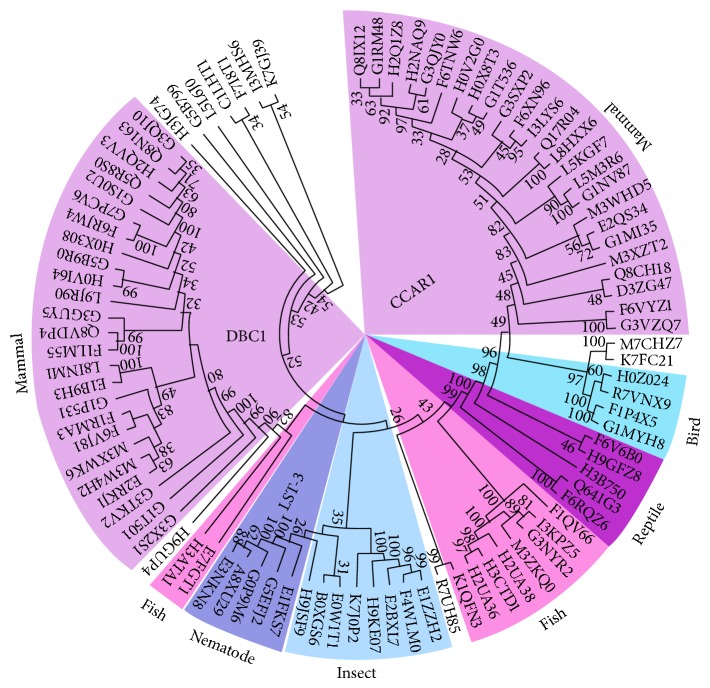
Phylogenetic analysis of DBC1 and CCAR1 homologs. A phylogenetic tree was built for all 93 protein sequences in 65 species as listed in Table  S1 using Mega5 software. All DBC1 proteins are on the left, while all CCAR1 proteins are on the right. The nematode LST-3 proteins, although closely related to CCAR1, are categorized into a subgroup of CCAR1. The colored shadows cover several regions that are extensively discussed in the paper.

To study the evolutionary relationship between DBC1 and CCAR1, phylogenetic analysis was performed with the DBC1 homologs and paralogs listed in Table  S1, and the results are shown in Figure 3. Most mammals have evolved to incorporate both DBC1 and CCAR1 into their genomes (Figure 3, purple shaded region). As a comparison, insects and nematodes have only incorporated CCAR1 into their genomes (Figure 3, light and dark blue shaded regions). Interestingly, the first known evolutionary appearance of DBC1 is in zebrafish (Figure 3, pink shaded region). This is a clear indication that DBC1 emerged later than CCAR1. Another interesting observation is that the nematode LST-3 proteins are more closely related to CCAR1 in lower species, such as insects. These observations have revealed an interesting evolutionary picture of DBC1/CCAR1/LST-3, where DBC1 has evolved from CCAR1 and CCAR1 originated from LST-3.

To compare homology between nematode LST-3 and hDBC1/hCCAR1, a cross-validation between the nematode proteome and the human proteome was performed. Specifically, the nematode* Caenorhabditis elegans* protein LST-3 was aligned against the complete human proteome and the only two significant hits (*E* value <1.0*e* − 20) were hDBC1 and hCCAR1. Conversely, both hDBC1 and hCCAR1 protein sequences were aligned against the complete* C. elegans* proteome, and the only significant hit was LST-3. This result shows that the only possible ancestor of DBC1 and CCAR1 in higher species is nematode LST-3.

**Figure 4:**
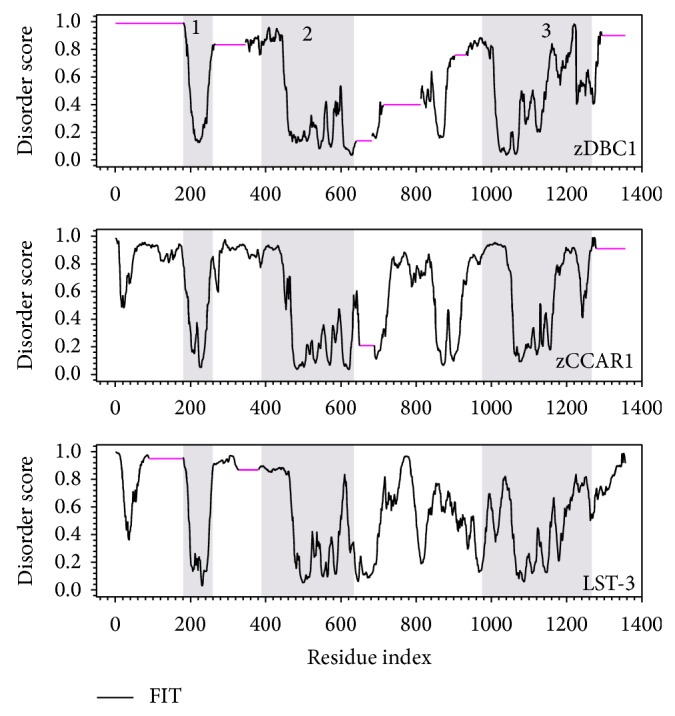
Gapped disorder prediction for zDBC1, zCCAR1, and LST-3. The disorder prediction was analyzed by PONDR-FIT for zebrafish DBC1 (zDBC1, UniProt ID: E7FGT1), zebrafish CCAR1 (zCCAR1, UniProt ID: F1QV66), and* C. elegans* LST-3 (UniProt ID: G5EFJ2). Residues with a score higher than 0.5 on the *y*-axis are disordered, while residues with score lower than 0.5 are structured. The *x*-axis represents the amino acid number. The black line represents the disorder prediction, while the pink horizontal lines represent gapped segments. The shaded regions represent similar patterns seen between all three proteins.

To further explore the evolutionary path of LST-3, CCAR1, and DBC1, we compared the full-length sequences of* C. elegans* LST-3, zebrafish CCAR1 (zCCAR1), and zebrafish DBC1 (zDBC1), as the first emergence of DBC1 is in zebrafish. The resulting gapped-disorder curve of this comparison is shown in Figure 4. A gapped disorder curve aligns proteins by the shape of the curve based on the disorder score. Instead of presenting the actual sequence similarity, the gapped disorder curve describes the overall similarity of the flexibility of the protein segments, which indicates any evolutionary gaps that may be present between proteins [72, 89, 90].

The structural similarities shared between zDBC1, zCCAR1, and LST-3 include a structured segment present at aa200 (Figure 4, shaded area 1), a similar disordered curvature spanning from aa200 to 400 (Figure 4, shaded area 2), the fluctuating peaks from aa500 to 600, and the increasingly disordered C-termini beginning at aa1100 and continuing to the end of the proteins (Figure 4, shaded area 3). The N-termini of zDBC1 and LST-3 have two gapped regions (pink lines) that are located immediately before and after the S1-Like domain. zCCAR1 contains two other gapped segments similar to zDBC1, one near the center of the protein that corresponds to the conserved Nudix domain and another on the C-terminus that does not correspond to a well-defined functional region. When compared to either zCCAR1 or LST-3, zDBC1 contains four additional gapped regions, thus indicating a difference of four insertions or deletions throughout evolution. The locations of these insertions and deletions are roughly in line with the gapped segments in the sequence alignment provided in Figures  S4 and  S5.

### 3.3. DBC1 Is More Conserved than CCAR1

**Figure 5:**
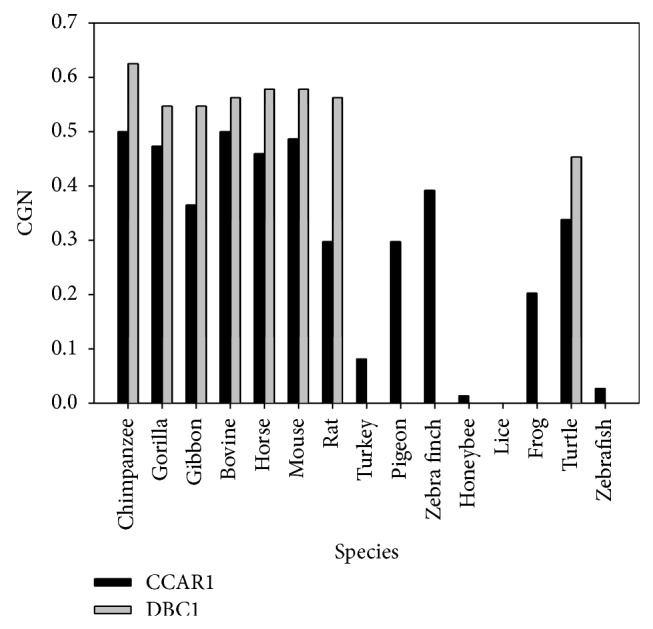
CGN score between human and other species for both CCAR1 (black) and DBC1 (gray). A high CGN score of >0.5 indicates that more than half of the gene neighbors are conserved within 2 Mb and shows conservation of the local chromosomal environment, while a score of <0.5 indicates that less than half of the neighboring genes are conserved.

The conservation of the genomic neighborhood (CGN) of DBC1 and CCAR1 was calculated for mammals, birds, insects, reptile, amphibians, and fish (Figure 5). The DBC1 gene is not present in birds, insects, or amphibians, but in the species where DBC1 gene is present, the CGN score of the DBC1 gene for that species is higher than that of CCAR1. This is another indication that the genomic region surrounding the CCAR1 gene is less conserved than the DBC1 gene.

**Figure 6:**
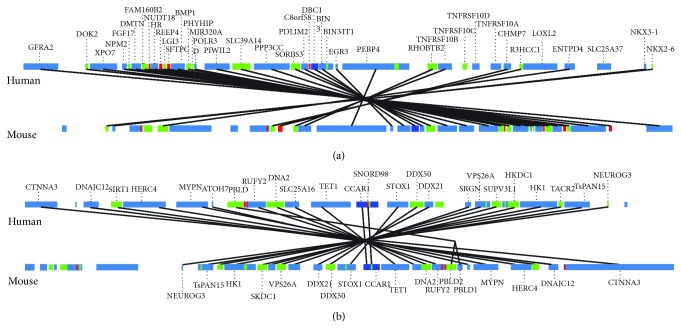
Genome neighborhood analysis between human and mouse DBC1 (a) and CCAR1 (b). The lines connect identical genes from human and mouse, surrounding 2 Mb of either DBC1 (a) or CCAR1 (b).

Two synteny plots comparing the human and mouse genes for DBC1 and CCAR1 are illustrated in Figure 6. In these plots, more conserved genes can be found in the neighboring region of DBC1 as compared to CCAR1, also indicating that DBC1 is more conserved than CCAR1.

### 3.4. CCAR1 Appeared before DBC1 in Evolution

**Figure 7:**
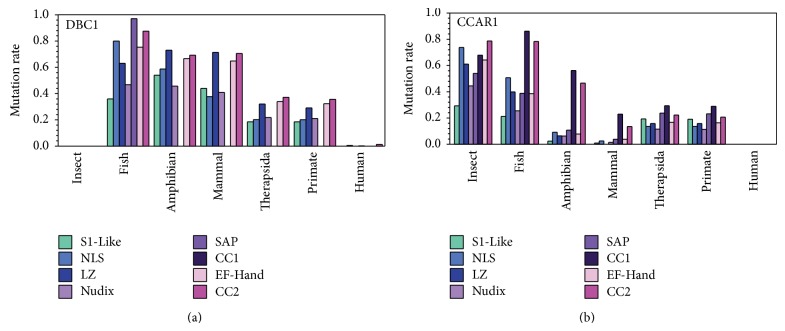
The amino acid substitution rates of specific domains among various groups of species for DBC1 (a) and CCAR1 (b). Based on the sequences in Table  S1, human is in one group, all other primates excluding human are in the second group, all other Therapsida excluding primates are in the third group, all other mammals compose the fourth group, amphibians are in the fifth group, fish are in the sixth group, and insects are in the seventh group. Eight domains were analyzed including the S1-Like, NLS, LZ, SAP, CC1, EF-Hand, and CC2 domains.

To further examine the variability of insertions and deletions in DBC1 and CCAR1, the amino acid substitution rate of each conserved domain across various groups of species was analyzed (Figure 7). The overall mutation rate for CCAR1 is approximately 20% from amphibian to human, while DBC1 has a relatively high substitution rate of about 50% from insect to mammal and 30% from Therapsida to primate. Even after DBC1 becomes more conserved after Therapsida, the substitution rate of various domains in DBC1 is still approximately 10% higher than that of the corresponding CCAR1 domains. Also, the mutation rate of each domain for DBC1 in fish is similar to that of insects for CCAR1, indicating a similar trend during the beginning of the evolutionary process between both proteins, with CCAR1 evolving first.

Examination of each domain individually reveals that the evolutionary process has varied between both proteins. Interestingly, the fish DBC1 protein contains a SAP domain that then disappears from amphibian and onward. The remaining S1-Like, NLS, LZ, Nudix, and CC2 domains continue to have high mutation rates from fish to amphibian, while the EF-Hand domain remains relatively conserved. Variation continues to be observed between all domains until a gradual steadying of mutation rates occurs in Therapsida and continues until human.

The mutation rate of each domain in CCAR1 exhibits a different trend. The mutation rate of the S1-Like, LZ, EF-Hand, SAP, NLS, and CC2 domains in CCAR1 varies from insects to fish, while the mutation rate between all domains decreases from fish to amphibian. Limited changes occur from amphibians to mammals in all domains except for the NLS and CC1 domains, where the mutation rate decreases more dramatically. This is followed by a further increase in mutation rates in all domains from mammals to Therapsida, with an eventual leveling off of the mutation rates in humans. This data further supports CCAR1 appearing first in evolution, not only by appearing in insects before DBC1, but also by becoming conserved much earlier in evolution.

### 3.5. DBC1 and CCAR1 Exhibit Similar Domain Flexibility

**Figure 8:**
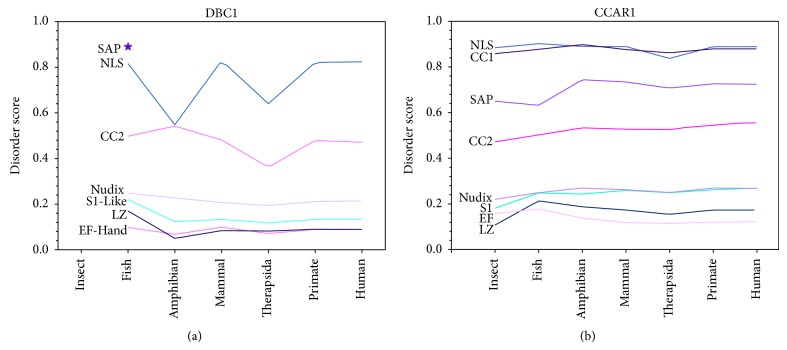
The average disorder score for each domain in DBC1 (a) and CCAR1 (b) across all grouped species. The categorization of domains and species groups is the same as in Figure 4. The disorder score for each domain is averaged for all of the sequences in each species group. The original disorder score was predicted by PONDR-FIT.

To determine if the mutation rate of each domain has affected protein structure and flexibility in the evolutionary process, the average disorder score of each domain across different species was compiled (Figure 8). The overall disorder scores between the various domains of DBC1 and CCAR1 are very similar. The NLS and CC2 domains are disordered in both proteins, while the Nudix, S1-Like, EF-Hand, and LZ are ordered domains. Also, the SAP domain in DBC1 that is only present in fish is highly disordered, corresponding with the intrinsic disorder of the SAP domain throughout CCAR1 evolution. Even though this overall similarity in structure exists, differences can be seen in the trend of intrinsic disorder across evolution between each domain in DBC1 and CCAR1. The S1-Like, Nudix, LZ, and EF-Hand domains in CCAR1 tend to become less structured throughout evolution, while in DBC1 the same domains become more structured.

During the evolutionary process, it appears that some domains underwent a drastic change in structural flexibility that is measured by predicted disorder score. In DBC1, the S1-Like and LZ domains decreased in structural flexibility from fish to amphibian, whereas the opposite trend is observed in CCAR1. The NLS and CC2 domains of DBC1 tend to drastically change in structural flexibility where a vibration pattern can be observed, before eventual leveling off into a disordered structure in primates, and carrying over into humans. In CCAR1, structural flexibility has increased in the SAP domain from fish to amphibian, The NLS domain had a sudden decrease in structural flexibility from mammals to Therapsida, before increasing from primate to human.

## 4. Discussion

DBC1 and CCAR1 are emerging as important regulators in a number of cellular pathways. DBC1 and CCAR1 share a similar domain structure, indicating they may have similar biological functions. The similar domain structure shared between the two multidomain proteins may also indicate origination from a common ancestor [91]. Therefore, it is important to investigate the process by which the functional domains of these two proteins have evolved. We predict here for the first time that DBC1 and CCAR1 are comprised of mostly intrinsically disordered regions, and that several of the functional domains in these two proteins are intrinsically disordered. These findings provide support for the role of these two proteins in many molecular interactions, as intrinsically disordered regions are frequent sites for protein-protein interactions [65]. In our prediction, the extended N-terminus of CCAR1 is much more flexible than that of DBC1, as denoted by a higher disorder score, which may indicate a unique functional role for this region. Corresponding to this, CCAR1 has recently been shown to have distinct functions apart from DBC1, such as binding directly to the LZ domain of DBC1 and synergistically regulating the function of DBC1 [14]. This regulatory ability would require a unique region capable of binding directly to DBC1, which our analysis suggests may be on the N-terminal domain.

It is often found that two protein paralogs develop throughout evolution from one common ancestor [92]. Supporting this notion, our analysis of DBC1 and CCAR1 from multiple species has established evidence that both proteins have evolved from one common nematode ancestor, LST-3. We have shown that CCAR1 first appears in insects, while DBC1 first appears much later in fish. Further supporting this claim is the fact that the DBC1 protein in fish is the only species in which DBC1 contains a SAP domain, a domain typically only found in CCAR1 proteins.

The SAP domain is a DNA/RNA binding domain with about 40 amino acids. The core structure of this domain is a two- or three-helix bundle. There are currently two X-ray structures of SAP domains in the PDB; however, in these two X-ray structures, the SAP domains are in complexes with other proteins and RNA. Therefore, these two X-ray structures of SAP domains cannot be used to assess the actual structural flexibility of the SAP domain by itself. There are also several other NMR structures for SAP domains in the PDB database. From these structures, it is clear that the SAP domain has huge structural flexibility. The RMSD values of structural alignment between these structures from different NMR experiments are very large. Therefore, results from NMR experiments provide evidence that the SAP domain is flexible. In addition, the length of the SAP domain is less than 40 amino acids, indicating that the SAP domain may not have enough hydrophobic interaction by itself. Further, in our prediction, it is clear that SAP domain is likely to be in a “dip” indicating a structure-prone tendency; however, this “dip” is flanked by two long disordered regions. Therefore, the possible 3D structural picture of the SAP domain is that this domain forms a small and flexible hydrophobic core and sits in the middle of a long disordered region. The SAP domain of LST-3 was likely passed on to the fish DBC1 protein in the early evolutionary history of DBC1. Other species may not have required this particular domain in DBC1 and hence continued to evolve without it. All of these findings combined suggest that LST-3 is the common ancestor and ortholog, to both DBC1 and CCAR1.

A gapped disorder plot shows that zDBC1 contains four additional gapped regions when compared to zCCAR1 and LST-3, indicating that a difference of four insertions/deletions and/or substitutions allowed for the first evolutionary appearance of DBC1 in zebrafish. As mutation rate is linked to evolution, understanding the mutation rates of these proteins can help to decipher their evolutionary history. We see that CCAR1 emerges earlier in evolution in insects and that it becomes relatively conserved by the amphibian period with the exception of the CC1 and the CC2 regions, which were still undergoing evolution. DBC1, on the other hand, took much longer to become relatively conserved, and still has not yet reached the low mutation rate of CCAR1, indicating the possibility of acquiring new functional roles in the future evolutionary period. DBC1 becomes relatively conserved in Therapsida. Taking into consideration that Therapsida appeared about 300 million years later than amphibians, this provides evidence that DBC1 arose much later in evolution than CCAR1, further supporting the notion that a CCAR1 homolog gave rise to DBC1 over time.

Domain-level analysis provides yet more information on the correlation between protein flexibility and evolution. The disorder scores for the NLS and CC2 domains in DBC1 have undergone drastic changes in the evolutionary process. This may indicate the sudden acquisition of a hydrophobic core, and thus an increased protein-binding ability. However, since this new function may not be essential for the species, the acquired hydrophobic binding sites may disappear in the evolutionary process.

The LZ domain of DBC1 has been implicated in a variety of regulatory processes. The LZ domain is a heptad repeat of leucine residues, which represents the hydrophobic core of a coiled-coil formed by two different chains. The basic regions next to the LZ domain along the coiled-coil can interact with the major groove of DNA to regulate the process of gene expression. A substitution in heptad repeats from hydrophobic to less-hydrophobic or hydrophilic and charged residues will lessen the hydrophobic interaction and distort the structure of the coiled-coil and thereby prevent DNA binding ability. Therefore, in order to keep the function of the LZ domain, all of the mutations on this domain should be hydrophobic-dominant and thus be more structure-prone with a lower disorder score. In DBC1, the LZ domain tends to become more structure-prone. Conversely, the LZ in CCAR1 loses structure slightly from insect to fish. It is interesting to see this deviation since DBC1 first appears in fish. The presence of DBC1 in fish may have resulted in a decreased requirement for the LZ domain in CCAR1, ultimately affecting the current functional role of the LZ in modern DBC1 and CCAR1. This same concept is also applicable to all of the structured domains as well.

Overall, our data presents new findings on the structure and evolution of DBC1 and CCAR1. Our findings support the function of both proteins in many protein-protein interactions due to the high occurrence of disordered residues. We have found an unstructured region on the N-terminus of CCAR1 that may be responsible for unique protein interactions independent of DBC1. Similarly, we have determined that the LZ of DBC1 may be involved in unique interactions, as the LZ of CCAR1 has become unstructured and possibly nonfunctional throughout evolution. We see that CCAR1 appeared much earlier in the process of evolution as compared to DBC1, and that the nematode LST-3 protein may be the common ancestor of DBC1 and CCAR1. As the nematode* C. elegans* is a model organism frequently used in experimental biology; this work may help to broaden DBC1 and CCAR1 studies by demonstrating the important role that nematodes have had in the evolution of these two proteins. Specifically, the nematode LST-3 protein may have undergone multiple insertions and deletions to give rise to modern-day CCAR1 and DBC1.

## Supplementary Material

Supplementary. Figure 1: Sequence alignment of DBC1 domains from various species.
Supplementary. Figure 2: Sequence alignment of CCAR1 domains from various species.
Supplementary. Figure 3: Alignment of C-ter domain between human DBC1 (hDBC1) and human CCAR1 (hCCAR1).
Supplementary. Figure 4: Sequence alignment between zebrafish CCAR1 (Uniprot ID: F1QV66) and C. elegans CCAR1 (Uniprot ID: G5EFJ2).
Supplementary. Figure 5: Sequence alignment between zebrafish CCAR1 (Uniprot ID: F1QV66) and zebrafish DBC1 (Uniprot ID: E9QH28).
Supplementary. Table 1: Lists of all DBC1, CCAR1, and LST-3 sequences used in the study and their Uniprot IDs.

## Figures and Tables

**Table 1:** The domain structure and function of human DBC1 and CCAR1. The domains for hDBC1 and hCCAR1 are depicted along with the amino acid boundaries for each domain. The known or predicted function of each of the conserved domains is listed.

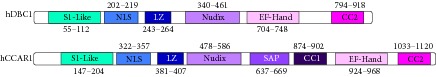	

	Domain Function

S1-Like^a,b^	Homology to an RNA-binding domain.
NLS^a,c^	Nuclear localization signal. Acetylation of the NLS in DBC1 regulates nuclear localization.
LZ^a,b,c,d^	Likely non-functional in CCAR1. Regulation of a diverse set of cellular pathways in DBC1.
Nudix^b^	Catalytically inactive hydrolase domain in DBC1 and CCAR1. Predicted to function as a sensor in DBC1 that may bind to NAD metabolites and regulate SIRT1.
*SAP*⁡^a,b,d^	Homology to a putative DNA-binding motif predicted to be involved in chromosomal organization.
EF-Hand^a,b^	Inactive variant of a calcium dependent regulator of multiple cellular processes.
CC^a,c,d^	Predicted protein-protein interaction motif.

Domain Boundary References: a: [8], b: [9], c: [14], d: [15].

## Abbreviations

DBC1: Deleted in breast cancer 1
CCAR1: Cell cycle and apoptosis regulator 1
LST-3: Lateral signaling target 3
hDBC1: Human DBC1
hCCAR1: Human CCAR1
zDBC1: Zebrafish DBC1
zCCAR1: Zebrafish CCAR1
CC1: Coiled-coil 1
CC2: Coiled-coil 2
NLS: Nuclear localization signal
LZ: Leucine zipper
SIRT1: Silent mating type information regulation 2 homolog
HDAC3: Histone deacetylase 3
aa: amino acid(s).
