# Supplementary material for: DBC1/CCAR2 and CCAR1 Are Largely Disordered Proteins that Have Evolved from One Common Ancestor
Source: Biomed Res Int. 2014 Dec 11;2014:418458. doi: 10.1155/2014/418458 (PMC4287135; doi:10.1155/2014/418458)
Supplement: Supplementary file 1 — Supplementary. Figure 1: Sequence alignment of DBC1 domains from various species. Supplementary. Figure 2: Sequence alignment of CCAR1 domains from various species. Supplementary. Figure 3: Alignment of C-ter domain between human DBC1 (hDBC1) and human CCAR1 (hCCAR1). Supplementary. Figure 4: Sequence alignment between zebrafish CCAR1 (Uniprot ID: F1QV66) and C. elegans CCAR1 (Uniprot ID: G5EFJ2). Supplementary. Figure 5: Sequence alignment between zebrafish CCAR1 (Uniprot ID: F1QV66) and zebrafish DBC1 (Uniprot ID: E9QH28). Supplementary. Table 1: Lists of all DBC1, CCAR1, and LST-3 sequences used in the study and their Uniprot IDs. [file 418458.f1.pdf]

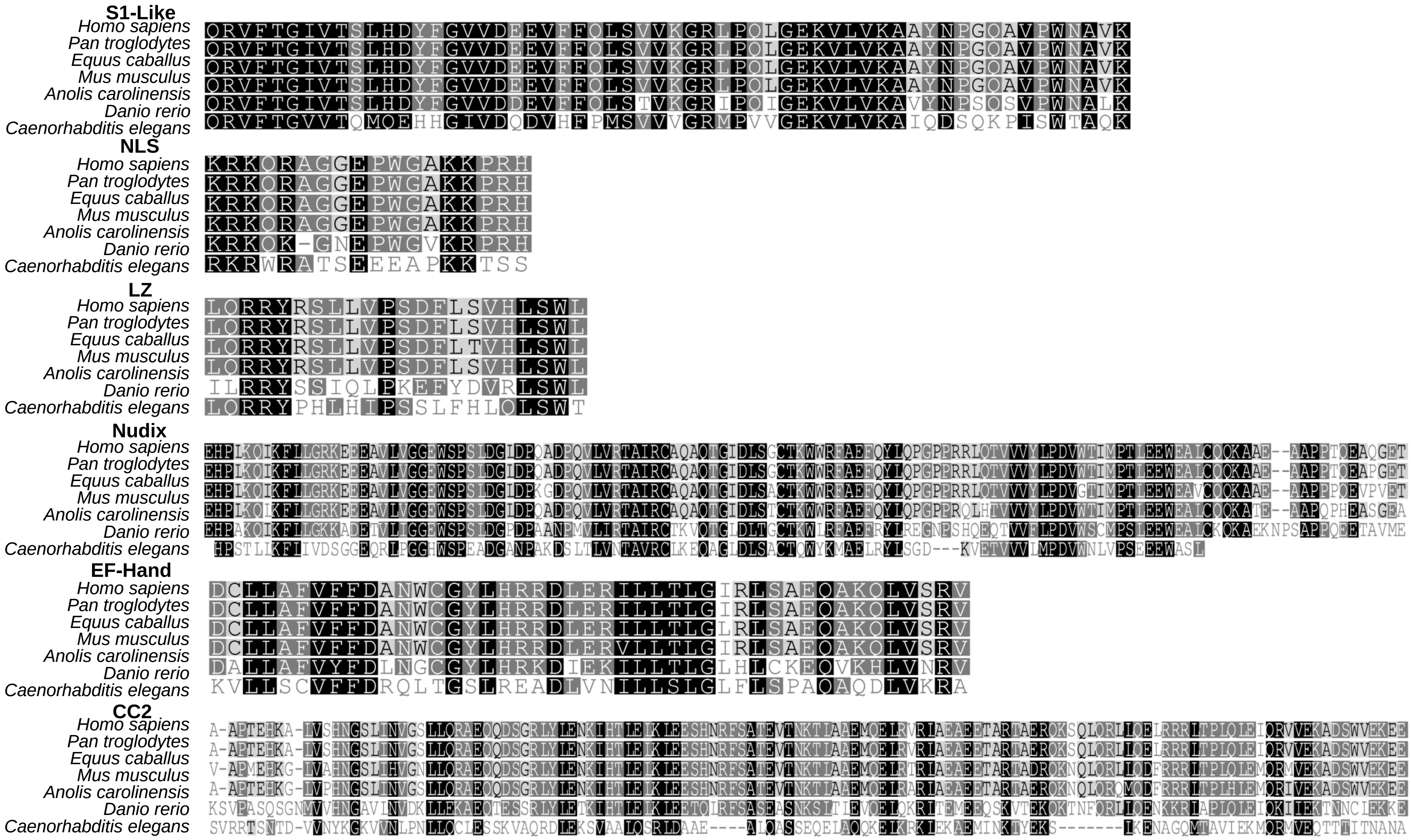
S1-Like
Homo sapiens
Pan troglodytes
Equus caballus
Mus musculus
Anolis carolinensis
Danio rerio
Caenorhabditis elegans
QRVFTGIVTSLHDYFGVVDEEVFFQLSVVKGRLPQLGEKVLVKAAYNPGQAVPWNAVK
QRVFTGIVTSLHDYFGVVDDEVFFQLSTVKGRIPQIGEKVLVKAVYNPSQSVPWNALK
QRVFTGVVTQMQEHHGIVDQDVHFPMSVVVGRMPVVGEKVLVKAIQDSQKPISWTAQK
NLS
KRKQRAGGEPWGAKKPRH
KRKQK-GNEPWGVKRPRH
RKRWRATSEEEAPKKTSS
LZ
LQRRYRSLLVPSDFLSVHLSWL
LQRRYRSLLVPSDFLTVHLSWL
ILRRYSSIQLPKEFYDVRLSWL
LQRRYPHLHIPSSLFHLQLSWT
Nudix
EF-Hand
DCLLAFVFFDANWCGYLHRRDLERILLTLGIRLSAEQAKQLVSRV
DCLLAFVFFDANWCGYLHRRDLERILLTLGLRLSAEQAKQLVSRV
DCLLAFVFFDANWCGYLHRRDLERVLLTLGIRLSAEQAKQLVSRV
DALLAFVYFDLNGCGYLHRKDIEKILLTLGLHLCKEQVKHLVNRV
KVLLSCVFFDRQLTGSLREADLVNILLSLGLFLSPAQAQDLVKRA
CC2
