# Supplementary material for: DBC1/CCAR2 and CCAR1 Are Largely Disordered Proteins that Have Evolved from One Common Ancestor
Source: Biomed Res Int. 2014 Dec 11;2014:418458. doi: 10.1155/2014/418458 (PMC4287135; doi:10.1155/2014/418458)
Supplement: Supplementary file 2 [file 418458.f2.pdf]

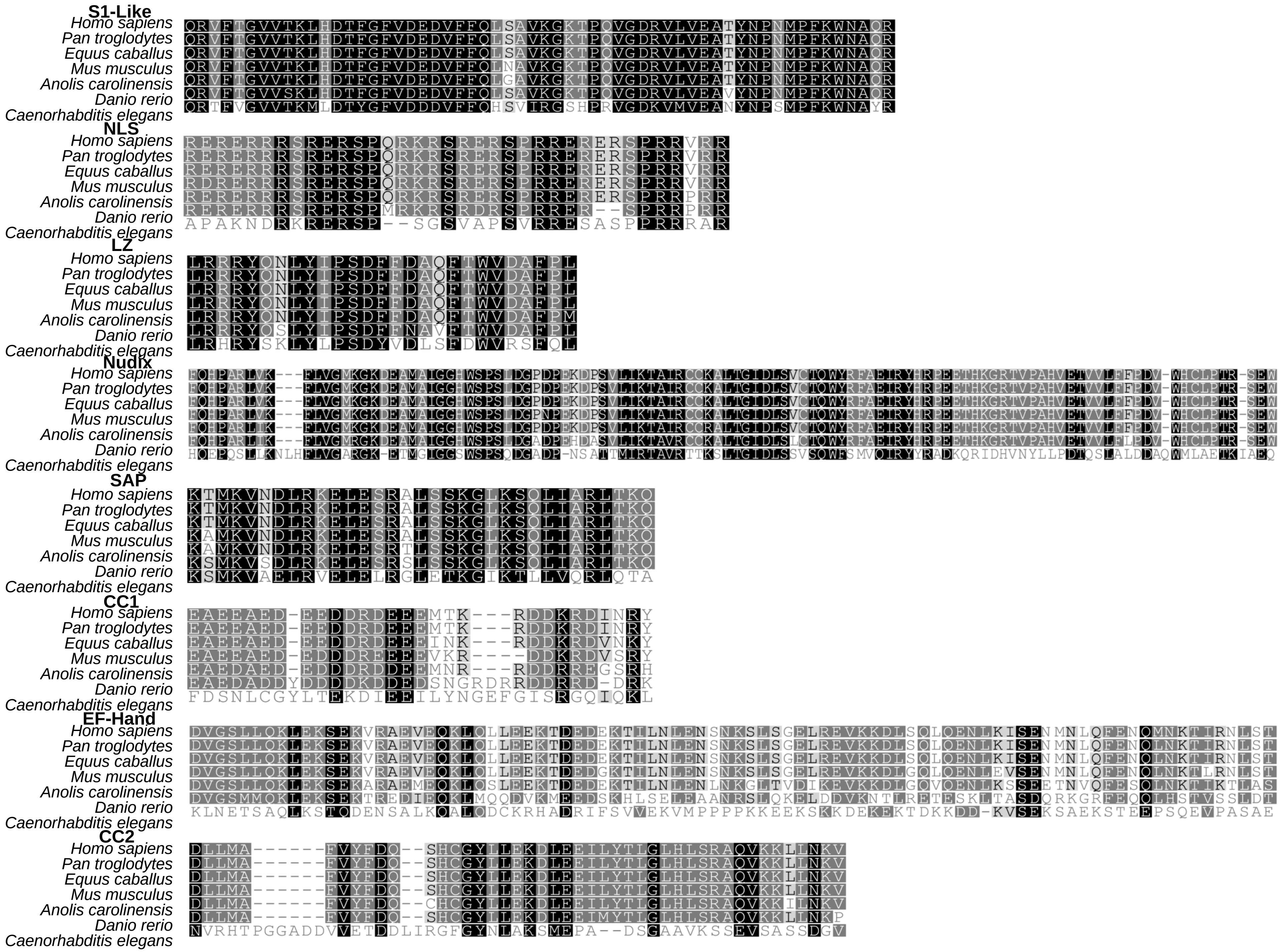

S1-Like
Homo sapiens QRVFTGVVTKLHDTFGFVDEDVFFQLSAVKGKTPQVGDRVLVEATYNPNMPFKWNAQR
Pan troglodytes QRVFTGVVTKLHDTFGFVDEDVFFQLSAVKGKTPQVGDRVLVEATYNPNMPFKWNAQR
Equus caballus QRVFTGVVTKLHDTFGFVDEDVFFQLSAVKGKTPQVGDRVLVEATYNPNMPFKWNAQR
Mus musculus QRVFTGVVTKLHDTFGFVDEDVFFQLNAVKGKTPQVGDRVLVEATYNPNMPFKWNAQR
Anolis carolinensis QRVFTGVVTKLHDTFGFVDEDVFFQLGAVKGKTPQVGDRVLVEATYNPNMPFKWNAQR
Danio rerio QRVFTGVVSKLHDTFGFVDEDVFFQLSAVKGKTPQVGDRVLVEAVYNPNMPFKWNAQR
Caenorhabditis elegans QRTFVGVVTKMLDTYGFVDDDVFFQHSVIRGSHPRVGDKVMVEANYNPSMPFKWNAYR
NLS
Homo sapiens RERERRRSRERSPQRKRSRERSPRRERERSPRRVRR
Pan troglodytes RERERRRSRERSPQRKRSRERSPRRERERSPRRVRR
Equus caballus RERERRRSRERSPQRKRSRERSPRRERERSPRRVRR
Mus musculus RDRERRRSRERSPQRKRSRERSPRRERERSPRRVRR
Anolis carolinensis RERERRRSRERSPQRKRSRERSPRRERERSPRRPRR
Danio rerio RERERRRSRERSPMRKRSRDRSPRRER--SPRRPRR
Caenorhabditis elegans APAKNDRKRERSP--SGSVAPSVRRESASPPRRRAR
LZ
Homo sapiens LRRRYQNLYIPSDFFDAQFTWVDAFPL
Pan troglodytes LRRRYQNLYIPSDFFDAQFTWVDAFPL
Equus caballus LRRRYQNLYIPSDFFDAQFTWVDAFPL
Mus musculus LRRRYQNLYIPSDFFDAQFTWVDAFPL
Anolis carolinensis LRRRYQNLYIPSDFFDAQFTWVDAFPM
Danio rerio LRRRYQSLYIPSDFFNAVFTWVDAFPL
Caenorhabditis elegans LRHRYSKLYLPSDYVDLSFDWVRSFQL
Nudix
Homo sapiens FQHPARLVK---FLVGMKGKDEAMAIGGHWSPSLDGPDPEKDPSVLIKTAIRCCKALTGIDLSVCTQWYRFAEIRYHRPEETHKGRTVPAHVETVVLFFPDV-WHCLPTR-SEW
Pan troglodytes FQHPARLVK---FLVGMKGKDEAMAIGGHWSPSLDGPDPEKDPSVLIKTAIRCCKALTGIDLSVCTQWYRFAEIRYHRPEETHKGRTVPAHVETVVLFFPDV-WHCLPTR-SEW
Equus caballus FQHPARLVK---FLVGMKGKDEAMAIGGHWSPSLDGPDPEKDPSVLIKTAIRCCKALTGIDLSVCTQWYRFAEIRYHRPEETHKGRTVPAHVETVVLFFPDV-WHCLPTR-SEW
Mus musculus FQHPARLVK---FLVGMKGKDEAMAIGGHWSPSLDGPNPEKDPSVLIKTAIRCCKALTGIDLSVCTQWYRFAEIRYHRPEETHKGRTVPAHVETVVLFFPDV-WHCLPTR-SEW
Anolis carolinensis FQHPARLIK---FLVGMKGKDEAMAIGGHWSPSLDGPDPEKDPSVLIKTAIRCCRALTGIDLSVCTQWYRFAEIRYHRPEETHKGRTVPAHVETVVLFFPDV-WHCLPTR-SEW
Danio rerio FQHPARLIK---FLVGMRGKDEAMAIGGHWSPSLDGADPEHDASVLIKTAVRCCKALTGIDLSLCTQWYRFAEIRYHRPEETHKGRTVPAHVETVVLFLPDV-WHCLPTR-SEW
Caenorhabditis elegans HQEPQSLLKNLHFLVGARGK-ETMGIGGSWSPSQDGADP-NSATTMIRTAVRTTKSLTGIDLSSVSQWFSMVQIRYYRADKQRIDHVNYLLPDTQSLALDDAQWMLAETKIAEQ
SAP
Homo sapiens KTMKVNDLRKELESRALSSKGLKSQLIARLTKQ
Pan troglodytes KTMKVNDLRKELESRALSSKGLKSQLIARLTKQ
Equus caballus KTMKVNDLRKELESRALSSKGLKSQLIARLTKQ
Mus musculus KAMKVNDLRKELESRALSSKGLKSQLIARLTKQ
Anolis carolinensis KAMKVNDLRKELESRTLSSKGLKSQLIARLTKQ
Danio rerio KSMKVSDLRKELESRSLSSKGLKSQLIARLTKQ
Caenorhabditis elegans KSMKVAELRVELELRGLETKGIKTLLVQRLQTA
CC1
Homo sapiens EAEEAED-EEDDRDEEEMTK---RDDKRDINRY
Pan troglodytes EAEEAED-EEDDRDEEEMTK---RDDKRDINRY
Equus caballus EAEEAED-EEDDRDEEEINK---RDDKRDVNKY
Mus musculus EAEEAED-EDDDREEEEVKR----DDKRDVSRY
Anolis carolinensis EAEDAED-EDDDRDDEEMNR---RDDRREGSRH
Danio rerio EAEDADDYDDDDKDDEDSNGRDRRDDRRD-DRK
Caenorhabditis elegans FDSNLCGYLTEKDIEEILYNGEFGISRGQIQKL
EF-Hand
Homo sapiens DVGSLLQKLEKSEKVRAEVEQKLQLLEEKTDEDEKTILNLENSNKSLSGELREVKKDLSQLQENLKISENMNLQFENQMNKTIRNLST
Pan troglodytes DVGSLLQKLEKSEKVRAEVEQKLQLLEEKTDEDEKTILNLENSNKSLSGELREVKKDLSQLQENLKISENMNLQFENQLNKTIRNLST
Equus caballus DVGSLLQKLEKSEKVRAEVEQKLQLLEEKTDEDEKTILNLENSNKSLSGELREVKKDLSQLQENLKISENMNLQFENQLNKTIRNLST
Mus musculus DVGSLLQKLEKSEKVRAEVEQKLQLLEEKTDEDGKTILNLENSNKSLSGELREVKKDLGQLQENLEVSENMNLQFENQLNKTLRNLST
Anolis carolinensis DVGSLLQKLEKSEKARAEMEQKLQSLEEKTDEDEKTILNLENLNKGLTVDIKEVKKDLGQVQENLKSSEETNVQFESQLNKTIKTLAS
Danio rerio DVGSMMQKLEKSEKTREDIEQKLMQQDVKMEEDSKHLSELEAANRSLQKELDDVKNTLRETESKLTASDQRKGRFEQQLHSTVSSLDT
Caenorhabditis elegans KLNETSAQLKSTQDENSALKQALQDCKRHADRIFSVVEKVMPPPPKKEEKSKKDEKEKTDKKDD-KVSEKSAEKSTEEPSQEVPASAE
CC2
Homo sapiens DLLMA------FVYFDQ--SHCGYLLEKDLEEILYTLGLHLSRAQVKKLLNKV
Pan troglodytes DLLMA------FVYFDQ--SHCGYLLEKDLEEILYTLGLHLSRAQVKKLLNKV
Equus caballus DLLMA------FVYFDQ--SHCGYLLEKDLEEILYTLGLHLSRAQVKKLLNKV
Mus musculus DLLMA------FVYFDQ--SHCGYLLEKDLEEILYTLGLHLSRAQVKKLLNKV
Anolis carolinensis DLLMA------FVYFDQ--CHCGYLLEKDLEEILYTLGLHLSRAQVKKILNKV
Danio rerio DLLMA------FVYFDQ--SHCGYLLEKDLEEIMYTLGLHLSRAQVKKLLNKP
Caenorhabditis elegans NVRHTPGGADDVETDDLIRGFGYNLAKSMEPA--DSGAAVKSSEVSASSDGV
