# Supplementary material for: DBC1/CCAR2 and CCAR1 Are Largely Disordered Proteins that Have Evolved from One Common Ancestor
Source: Biomed Res Int. 2014 Dec 11;2014:418458. doi: 10.1155/2014/418458 (PMC4287135; doi:10.1155/2014/418458)
Supplement: Supplementary file 3 [file 418458.f3.pdf]

```
hDBC1-C    481   NAETPEATTQQETDTDLPEAPPPPLEPAVIARPGCVNLSLHGIVEDRRPKER-ISFEVMV
hCCAR1-C   721   -----------YILPDEPAIIVHPNWAAKSGKFDCSIMSLSVLLDYRLEDNKEHSFEVSL
                               .* *     *  .*  .: .*  :**  ::: *  .::  **** :
hDBC1-C          LAELFLEMLQRDFGYRVYKMLLSLPEKVVSPPEPEKEEAKEEATKEEEAIKEEVVKEPK
hCCAR1-C         FAELFNEMLQRDFGVRIYKSLLSLPEKED---KKEKDKKSKKDERKDKKEERDDETDEPK
                 :**** ******** *:** *******     : **:: :*::  *:::  ::: ..***
hDBC1-C          DEAQNEGPATESEAPLKEDGLLPKPLSSGGEEEEKPRGEASEDLCEMALDPELLLLRDDG
hCCAR1-C         PKRRKSGDDKDKKEDRDERKKEDKRKDDSKDDDETEEDNNQDEYDPMEAEEAEDEEDDRD
                  : ::.*  .:.:   .*     *  ... :::*. ..: .::   *  :       * .
hDBC1-C          EEEFAGAKLEDSEVRSVASNQSEMEFSSLQDMPKELDPSAVLPLDCLLAFVFFDANWCGY
hCCAR1-C         EEEMTKRDDKRDINRYCKERPS-------KDKEKEKTQMITINRDLLMAFVYFDQSHCGY
                 ***::  . : .  *   .. *       :*  **     .:  * *:***:** . ***
hDBC1-C          LHRRDLERILLTLGIRLSAEQAKQLVSRVVTQNICQYRSLQYSRQEGLDGGLP---EEVL
hCCAR1-C         LLEKDLEEILYTLGLHLSRAQVKKLLNKVVLRESCFYRKLTDTSKDEENHEESESLQEDM
                 * .:***.** ***::**  *.*:*:.:** :: * **.*  : ::  :   .   :* :
hDBC1-C          FGNLDLLPPPGKSTKPGAAPTEHKALVSHNGSLINVGSLLQRAEQQDSGRLYLENKIHTL
hCCAR1-C         LGNRLLLPTP-TVKQESKDVEENVGLIVYNGAMVDVGSLLQKLEKSEKVRAEVEQKLQLL
                 :**  ***.* . .: .    *: .*: :**::::******: *:.:. *  :*:*:: *
hDBC1-C          ELKLEESHNRFSATEVTNKTLAAEMQELRVRLAEAEETARTAERQKSQLQRLLQELRRRL
hCCAR1-C         EEKTDEDEKTILNLENSNKSLSGELREVKKDLSQLQENLKISENMNLQFENQMNKTIRNL
                 * * :*..: :   * :**:*:.*::*::  *:: :*. : :*. : *::. :::   *.*
hDBC1-C          TPLQLEIQRVVEKADSWVEKEEPAPSN-----      923
hCCAR1-C         STVMDEIHTVLKKDNVKNEDKDQKSKENGASV     1150
                 :.:  **: *::* :   *.::  ..:
```
