# Supplementary material for: DBC1/CCAR2 and CCAR1 Are Largely Disordered Proteins that Have Evolved from One Common Ancestor
Source: Biomed Res Int. 2014 Dec 11;2014:418458. doi: 10.1155/2014/418458 (PMC4287135; doi:10.1155/2014/418458)
Supplement: Supplementary file 4 [file 418458.f4.pdf]

```
F1QV66          MAQFGGQKNPPWAAQFAATAVSQPGHTGQSLDLNSLHSLGVQQQSLLGASPMYTQQSALA 60
G5EFJ2          MSQFGGGKPP--------GQWHRPAAPGGAPQLSGFSAVNFGGVPMVGMG---VPMGMVN 49
                *:**** * *              :*. .* : :*..: ::..   .::* .   .  . :
F1QV66          AASLNSQSAANYQLSQQTAALQQQAAAAAAAAALQQSQINSALQQYQQQQQQQQQPPQAP 120
G5EFJ2          QAAFSSQGVLGMQG---------------IMGAVPQQSLPQQQPNFQQQNSLQQG----- 89
                 *::.**.. . *                .*: *..: .   ::***:. **
F1QV66          PPQPPPQQTLYNVPHQLPQPQQALLSQPPVALPTSLSLSNPQQTAQITVSYPTPRSSHQQ 180
G5EFJ2          -------------------------------------------------------TNMQQ 94
                                                                       :. **
F1QV66          QTQPQKQRVFTGVVSKLHDTFGFVDEDVFFQLSAVKGKTPQVGDRVLVEAVYNPNMPFKW 240
G5EFJ2          NSGAKNQRTFVGVVTKMLDTYGFVDDDVFFQHSVIRGSHPRVGDKVMVEANYNPSMPFKW 154
                :: .::**.*.***:*: **:****:***** *.::*. *:***:*:*** ***.*****
F1QV66          NAQRIQTLPQLPNQTHQPPQPLPQVSPQLSSFYTDAGMQRYSDLHSAVDSRQNSQPQVPN 300
G5EFJ2          NAYRIQLLNAATQQ-----EPARQAPQQQQMHQPQRGGQESQRWGAAPASGTSDRADRNG 209
                ** *** *   .:*      :*  *.. * . . .: * *. .   :*  *  ..:.:  .
F1QV66          MMKPGPTMLQSLPPPTTFSVPAQGPPPSLLQAQLSAASLAPLLQNPPQPLLPQPPPKDSV 360
G5EFJ2          GARGNDSPLHRSSAARRHSPPPRRASP---------------------PRRTSSPKRDAR 248
                  : . : *:  ...  .* *.: ..*                     *  ...* :*:
F1QV66          FSGGLLQPPVRMMPQPQQVRRVEPSPRFPNRSDRPELILRKDDRSRERERERRRSRERSP 420
G5EFJ2          PAR-----EIRDSREPREVRRSPP----PRRAASP-----RKASSPSAPAKNDRKRERSP 294
                 :       :*    :*::***  *     *.*:  *      :.  * .   :. *.*****
F1QV66          MRKRSRDRSPRRERSPRRPRRVVPRYTVQFSKFSLDGYNCDMMELRRRYQSLYIPSDFFN 480
G5EFJ2          SGSVAPSVRRESASPPRRRARIIPRYECRAQKPALLSPIVSGSVLRHRYSKLYLPSDYVD 354
                 . : .   .   .***  *::***  : .* :* .   .   **:**..**:***:.:
F1QV66          AVFTWVDAFPLSRPFTFGNYCNFHIMHKEVDSLVKN-TAVLDPPDANHTYSAKVMLLANP 539
G5EFJ2          LSFDWVRSFQLDLSLDLSNPIQFHVFNKDVDFIGEEEIQDLDPEDADHRHQVKVLLLSHA 414
                  * ** :* *. .: :.*  :**:::*:** : ::    *** **:* :..**:**::.
F1QV66          SLDELYHKSCALSED-PAELRDSFQHPARLIKFLVGMRGKDEAMAIGGHWSPSLDGADPE 598
G5EFJ2          GKSEVVKKAFCLMADGTTDDHQEPQSLLKNLHFLVGARGK-ETMGIGGSWSPSQDGADPN 473
                . .*: :*: .*   *  .:: ::. *    : ::**** *** *:*.*** **** *****:
F1QV66          HDASVLIKTAVRCCKALTGIDLSLCTQWYRFAEIRYHRPEETHKGRTVPAHVETVVLFLP 658
G5EFJ2          S-ATTMIRTAVRTTKSLTGIDLSSVSQWFSMVQIRYYRADKQRIDHVNYLLPDTQSLALD 532
                  *:.:*:**** *:*******  :**: :.:***:*.:: : .:.      :*  * *
F1QV66          DVWHCLPTRSEWEELSRGLK---------------------------------EQLAEKLL 686
G5EFJ2          DAQWMLAETKIAEQLKAKLANVDALKIEEDEPPVVMMVEESESVVAAAAAAADVVPEQSI 592
                *.   *.  .  *:*.  *                                : :.*: :
F1QV66          AERKEADGEQEEEDKDEDDSKEVTT---------------------------PTHWSKLD 719
G5EFJ2          PDVKKEEELQAEEPKVLDNVKAEESDVVADVSMNSTTDADNSEAPAAENGQGPTNWSNLD 652
                .: *: :  * ** *  *: *   :                           **:**:**
F1QV66          PKSMKVSDLRKELESRSLSSKGLKSQLIARLTKQLKVEEQVEESKEPEKPEPPSVEE--- 776
G5EFJ2          PKSMKVAELRVELELRGLETKGIKTLLVQRLQTALDTEKAAEASVAARDVEMRDAAENAV 712
                ******::** *** *.*.:**:*: *: ** . *..*: .* *   ... *  .. *
F1QV66          -------------------------DESCRLEDDREEEERKRQEEQERQRRERR------Y 806
G5EFJ2          KQEGGEENPAAFIAPSIEETKAKTEAEAKKEAEEAEKRKKKEEQLEKEKKEKREALEKHY 772
                                         : ..: * :. *:.:*::*: *::::*:*      *
F1QV66          VLPDEPTIIVHPNWAAKNGKFDCSIMSLSVLLDYRLEDNKEHSFEVSLFAELFNEMLQRD 866
G5EFJ2          QLPKDKKILVFPSKSFKSGKFDCKVLSLSSLLDYRHDDNKENQFEVSLFAEAFKEMIERN 832
                 **.: .*:*.*. : *.*****.::*** ***** :****:.******** *:**::*:
F1QV66          FGYRIYKALASLPTKDEKKDKKERAKKEAERRDIKKERDEDNGEPVAKRIREEDDKRKDE 926
G5EFJ2          AAFTIYETLANCGDRDAEK-------------------------------------KRRDE 856
                 .: **::**.   :* :*                                      **:**
F1QV66          EKERGIKREESKDDDDNEDGSSNNNADEYDPLEAEDADDYDDDDKDDEDSNGRDRRDDRR 986
G5EFJ2          AREKPVEPKEPKE-----------------PAETTEKAAEGEEKKDEKEEKRDEKKDEKR 899
                 :*: :: :*.*:                  * *: :    .::.**:::.:  :::*::*
F1QV66          DDRKSKDRSSKDKDEKKRQMVTFNKDLLMAFVYFDQSHCGYLLEKDLEEIMYTLGLHLSR 1046
G5EFJ2          EEKREK------VERIDLKSVVANRTVYEAFSLFDSNLCGYLTEKDIEEILYNGEFGISR 953
                ::::.*       :. . : *. *: :  **  **.. **** ***:***:*.  : :**
F1QV66          AQVKKLLNKPLVKESCHYRKLTDRPKDEPCPALISEAHIDNLLGNQILLT---------- 1096
G5EFJ2          GQIQKLAKKLSVRDKINYRHLTDVLTDMDGNVRHTPGGADDVVETDDLIRGFGYNLAKSM 1013
                .*::** :*  *::. :**:***  .*     .  : .  *::: .: *:
F1QV66          ----SQKIKREPDESGESGSLIVYKGALVDVGSMMQKLEKSEKTREDIEQKLMQQDVKME 1152
G5EFJ2          EPADSGAAVKSSEVSASSDGVVIINGSAVNVVQKMKLLKQVEKERDEAKSTVSEQLSLIE 1073
                    *    :..: *..*..::: :*: *:* . *: *:: ** *:: :..: :*   :*
F1QV66          EDSKHLSELEAANRSLQKELDDVKNTLRETESKLTASDQRKGRFEQQLHSTVSSLDTIKK 1212
G5EFJ2          QLREAKAEIDKKKKDIDSHYHKSNKKLNETSAQLKSTQDENSALKQALQDCKRHADRIFS 1133
                :  :  :*::  ::.::.. .. ::.*.**.::*.::::.:. ::* *:.     * * .
F1QV66          ELQGVLANNDHSEDADHKTQANGSDE---------------------------------- 1238
G5EFJ2          VVEKVMPPPPKKEEKSKKDEKEKTDKKDDKVSEKSAEKSTEEPSQEVPASAEQAAEPSTE 1193
                 :: *:.   :.*: .:* : : :*:
F1QV66          ----------------------
G5EFJ2          AEPIVIDESEDKDAEVVEESKE 1215
```
