# Supplementary material for: DBC1/CCAR2 and CCAR1 Are Largely Disordered Proteins that Have Evolved from One Common Ancestor
Source: Biomed Res Int. 2014 Dec 11;2014:418458. doi: 10.1155/2014/418458 (PMC4287135; doi:10.1155/2014/418458)
Supplement: Supplementary file 5 [file 418458.f5.pdf]

```
F1QV66          MAQFGGQKNPPWAAQFAATAVSQPGHTGQSLDLNSLHSLGVQQQSLLGASPMYTQQSALA 60
E9QH28          ------------------------------------------------------------

F1QV66          AASLNSQSAANYQLSQQTAALQQQAAAAAAAAALQQSQINSALQQYQQQQQQQQQPPQAP 120
E9QH28          ------------------------------------------------------------

F1QV66          PPQPPPQQTLYNVPHQLPQPQQALLSQPPVALPTSLSLSNPQQTAQITVSYPTPRSSHQQ 180
E9QH28          ------------------------------------------------------------

F1QV66          QTQPQKQRVFTGVVSKLHDTFGFVDEDVFFQLSAVKGKTPQVGDRVLVEAVYNPNMPFKW 240
E9QH28          METQMRQRVFTGVVTQMQEHHGIVDQDVHFPMSVVVGRMPVVGEKVLVKAIQDSQKPISW 60
                     :********::::: .*:**:**.* :*.* *: * **::***:*: :.: *:.*
F1QV66          NAQRIQTLPQLPNQTHQPPQPLPQVSPQLSSFYTDAGMQRYSDLHSAVDSRQNSQPQVPN 300
E9QH28          TAQKVQTLNGQPFKSPPP-----------------------------------LLPSMSS 85
                .**::***   * ::  *                                    *.:..
F1QV66          MMKPGPTMLQSLPPPTTFSVPAQGPPPSLLQAQLSAASLAPLLQNPPQPLLPQPPPKDSV 360
E9QH28          TLKPG-----------------------------------ILGTKPQPLLKSPKIPPLI 109
                 :***                                    :* . ***** .*     :
F1QV66          FSGGLLQPPVRMMPQPQQVRRVEPSPRFPNRSDRPELILRKDDRSRERERERRRSRERSP 420
E9QH28          PSMQPNSGGMIQLSHHQQMGWNGPFDGWGGGRKRHSEGMGGRRAGRWEDGGGGLWGGDLM 169
                 *    .  :  :.: **:    *    :  .   .* .  :     .* .:
F1QV66          MRKRSRDRSPRRERSPRRPRRVVPRYTVQFSKFSLDGYNCDMMELRRRYQSLYIPSDFFN 480
E9QH28          HQKRKRWRATSEEEAPKKTSSAATHSVPLFSCFSRDTQACDYLELQRRYPHLHIPSSLFH 229
                 :**.* *:. .*.:*::.  ...: .  ** ** *   ** :**:***  *:***.:*:
F1QV66          AVFTWVDAFPLSRPFTFGNYCNFHIMHKEVDSLVKNTAVLDPPDANHTYSAKVMLLANPS 540
E9QH28          LQLSWTESFPLDQPLPLRGPCLFHIGPNQPETEADVCESTD-----DAFAVRVLLFSMPC 284
                  ::*.::***.:*:.: . * ***  :: :: ..     *     .:::.:*:*:: *.
F1QV66          LDELYHKSCALSEDPAELRDSFQHPARLIKFLVGMRGKDEAMAIGGHWSPSLDGADPEHD 600
E9QH28          LEDVYSQCCNLSND-GQTQKEAVHPSTLLKFLIVDSG-GEQRLPGGHWSPEADGANPAKD 342
                *:::* :.* **:* .: :..  **: *:***:   * .*    ******. ***:* :*
F1QV66          ASVLIKTAVRCCKALTGIDLSLCTQWYRFAEIRYHRPEETHKGRTVPAHVETVVLFLPDV 660
E9QH28          SLTLVNTAVRCLKEQAGLDLSACTQWYKMAELRYLSGD----------KVETVVVLMPDV 392
                : .*::***** *  :*:*** *****::**:**   :          :*****:::***
F1QV66          WHCLPTRSEWEELSRGLKEQLAEKLLAERKEADGEQEEEDKDEDDSKEVTTPTHWSKLDP 720
E9QH28          WNLVPSEEEWASLQ---------------------------------------------- 406
                *: :*:..** .*.
F1QV66          KSMKVSDLRKELESRSLSSKGLKSQLIARLTKQLKVEEQVEESKEPEKPEPPSVEEDESC 780
E9QH28          ------------------------------------------------------------

F1QV66          RLEDDREEEERKRQEEQERQRRERRYVLPDEPTIIVHPNWAAKNGKFDCSIMSLSVLLDY 840
E9QH28          -LEDDLS--------------------LPESPSVVFHP-----SAGLNLSAVSLSSLLEP 440
                 **** .                    **:.*:::.**     .. :: * :*** **:
F1QV66          RLEDNKEHSFEVSLFAELFNEMLQRDFGYRIYKALASLPTKDEKKDKKERAKKEAERRDI 900
E9QH28          QTLQTR-DSCEVSLIAEMFSEMLQRDFGLQLYRCLCSLPQNISDPQTEAKQDNNTAKEEE 499
                :  :.: .* ****:**:*.******** ::*:.*.*** : .. :.: : .::: :.:
F1QV66          KKERDEDNGEPVAKRIREEDDKRKDEEKERGIKREESKDDDDNEDGSSNNNADEYDPLEA 960
E9QH28          EEIK--------------KSDKKTTKKKDSSDARKTVKEEKDAEDAMLTDETEAVGKQPS 545
                :: :              :.**:. ::*: .  *:  *::.* **.  .::::  .   :
F1QV66          EDADDYDDDDKDDEDSNGRDRRDDRRDDRKSKDRSSKDKDEKKRQMVTFNKDLLMAFVYF 1020
E9QH28          RDGKTVSAG-VEEQSSNAADN------------QTSKHCERPPGWTDELPRKVLLSCVFF 592
                .*..  . .  :::.**. *.            ::**. :.       : :.:*:: *:*
F1QV66          DQSHCGYLLEKDLEEIMYTLGLHLSRAQVKKLLNKPLVKESCHYRKLTDRPKDEPCPALI 1080
E9QH28          DRQLTGSLREADLVNILLSLGLFLSPAQAQDLVKRAAVGGLCLYRNLCSRWSDSAPSDAS 652
                *:.  * * * ** :*: :***.** **.:.*:::. *   * **:* .* .*.. .
F1QV66          SEAHIDNLLGNQILLTSQKIKREPDESGESGSLIVYKGALVDVGSMMQKLEKSEKTREDI 1140
E9QH28          AIAEGN----KAMLPTQPCKDRGSVRRTSNTDVVNYKGKVVNLPNLLQCLESSKVAQRDL 708
                : *. :    : :* *.   .* . .  .. .:: *** :*:: .::* **.*: ::.*:
F1QV66          EQKLMQQDVKMEEDSKHLSELEAANRSLQKELDDVKNTLRETESKLTASDQRKGRFEQQL 1200
E9QH28          EKSVAALQSRLD-----------AAEALQASSEQELAQQKELKRKLEKAEMINKTYEKSL 757
                *:.:   : :::           * .:** . ::     :* : **  ::  :  :*:.*
F1QV66          HSTVSSLDTIKKELQGVLANNDHSEDADHKTQANGSDE 1238
E9QH28          KENAGQMTAVIEKMQRMVEQTTTITNANAGKEEKL--- 792
                :.....: :: :::* :: :.    :*:  .: :
```
