# Supplementary material for: DBC1/CCAR2 and CCAR1 Are Largely Disordered Proteins that Have Evolved from One Common Ancestor
Source: Biomed Res Int. 2014 Dec 11;2014:418458. doi: 10.1155/2014/418458 (PMC4287135; doi:10.1155/2014/418458)
Supplement: Supplementary file 6 [file 418458.f6.pdf]

Species	DBC1	CCAR1	LST-3
Homo sapiens (Human)	Q8N163	Q8IX12	—
Pongo abelii (Orangutan)	Q5R8S0	H2NAQ9	—
Pan troglodytes (Chimpanzee)	H2QVV3	H2Q1Z8	—
Macaca mulatta (Rhesus Monkey)	F6RJW4	F6TNW6	—
Nomascus leucogenys (Gibbon)	G1S0U2	G1RM48	—
Gorilla gorilla gorilla (Lowland Gorilla)	G3QJ10	G3QJY0	—
Rattus norvegicus (Rat)	D3ZG47	F1LM55	—
Mus musculus (Mouse)	Q8VDP4	Q8CH18	—
Pteropus alecto (Black flying fox)	L5L6J0	L5KGF7	—
Spermophilus tridecemlineatus (Ground squirrel)	I3MHS6	I3LYS6	—
Mustela putorius furo (European domestic ferret)	M3XWK6	M3XZT2	—
Cavia porcellus (Guinea pig)	H0VI64	H0V2G0	—
Otolemur garnettii (Garnett’s greater bushbaby)	H0X308	H0X8T3	—
Sarcophilus harrisii (Tasmanian devil)	G3X2S1	G3VZQ7	—
Myotis lucifugus (Little brown bat)	G1P531	G1NV87	—
Felis catus (Cat)	M3W4H2	M3WHD5	—
Canis familiaris (Dog)	E2RKJ1	E2QS34	—
Ailuropoda melanoleuca (Giant panda)	G1L8L0	G1MI35	—
Loxodonta africana (African elephant)	G3TKV2	G3SXP2	—
Bos taurus (Bovine)	E1B9H3	Q17R04	—
Bos mutus (Wild yak)	L8INM1	L8HXX6	—
Equus caballus (Horse)	F6VJ81	F6XN96	—
Oryctolagus cuniculus (Rabbit)	G1T501	G1T536	—
Anolis carolinensis (Green anole)	H9GUP4	H9GFZ8	—
Pelodiscus sinensis (Chinese softshell turtle)	K7GJ39	K7FC21	—
Takifugu rubripes (Japanese pufferfish)	H2UA38	H2UA36	—
Latimeria chalumnae (West Indian ocean coelacanth)	H3ATA1	H3B750	—
Danio rerio (Zebrafish)	E7FGT1	F1QV66	—
Sus scrofa (Pig)	F1RMA3	—	—
Macaca fascicularis (Crab-eating macaque)	G7PCV6	—	—
Cricetulus griseus (Chinese hamster)	G3GUY5	—	—
Callithrix jacchus (White-tufted-ear marmoset)	—	F7I8T1	—
Tupaia chinensis (Chinese tree shrew)	—	L9JR90	—
Monodelphis domestica (Gray short-tailed opossum)	—	F6VYZ1	—
Myotis davidii (David’s myotis)	—	L5M3R6	—
Ornithorhynchus anatinus (Duckbill platypus)	—	F6V6B0	—
Columba livia (Domestic pigeon)	—	R7VNX9	—
Gallus gallus (Chicken)	—	F1P4X5	—
Taeniopygia guttata (Zebra finch)	—	H0Z024	—
Meleagris gallopavo (Common turkey)	—	G1MYH8	—
Xenopus laevis (African clawed frog)	—	Q641G3	—
Xenopus tropicalis (Western clawed frog)	—	F6RQZ6	—
Gasterosteus aculeatus (Three-spined stickleback)	—	G3NYR2	—
Xiphophorus maculatus (Southern platyfish)	—	M3ZKQ0	—
Tetraodon nigroviridis (Spotted green pufferfish)	—	H3CTD1	—
Oreochromis niloticus (Nile tilapia)	—	I3KPZ5	—

Chelonia mydas (Green sea-turtle)	—	M7CHZ7	—
Apis mellifera (Honeybee)	—	H9KE07	—
Nasonia vitripennis (Parasitic wasp)	—	K7J0P2	—
Culex quinquefasciatus (Southern house mosquito)	—	B0XGS6	—
Bombyx mori (Silk moth)	—	H9JSF9	—
Pediculus humanus subsp. corporis (Body louse)	—	E0W1T1	—
Camponotus floridanus (Florida carpenter ant)	—	E1ZZH2	—
Harpegnathos saltator (Jumping ant)	—	E2BXL7	—
Acromyrmex echinatior (Panamanian leafcutter ant)	—	F4WLM0	—
Crassostrea gigas (Pacific oyster)	—	K1QFN3	—
Strongylocentrotus purpuratus (Purple sea urchin)	—	H3JG74	—
Schistosoma japonicum (Blood fluke)	—	C1LHT1	—
Loa loa (Eye worm)		E1FKS7	
Caenorhabditis briggsae	—	—	A8XU29
Caenorhabditis brenneri (Nematode worm)	—	—	G0P9M6
Caenorhabditis elegans	—	—	G5EFJ2
Caenorhabditis remanei	—	—	E3NKN8
